# On pre-image iterations for speech enhancement

**DOI:** 10.1186/s40064-015-0983-x

**Published:** 2015-06-04

**Authors:** Christina Leitner, Franz Pernkopf

**Affiliations:** JOANNEUM RESEARCH Forschungsgesellschaft mbH, DIGITAL – Institute for Information and Communication Technologies, Steyrergasse 17, Graz, 8010 Austria; Graz University of Technology, Institute of Signal Processing and Speech Communication, Inffeldgasse 16c, Graz, 8010 Austria

**Keywords:** Speech enhancement, Speech de-noising, Kernel PCA, Automatic speech recognition

## Abstract

In this paper, we apply kernel PCA for speech enhancement and derive pre-image iterations for speech enhancement. Both methods make use of a Gaussian kernel. The kernel variance serves as tuning parameter that has to be adapted according to the SNR and the desired degree of de-noising. We develop a method to derive a suitable value for the kernel variance from a noise estimate to adapt pre-image iterations to arbitrary SNRs. In experiments, we compare the performance of kernel PCA and pre-image iterations in terms of objective speech quality measures and automatic speech recognition. The speech data is corrupted by white and colored noise at 0, 5, 10, and 15 dB SNR. As a benchmark, we provide results of the generalized subspace method, of spectral subtraction, and of the minimum mean-square error log-spectral amplitude estimator. In terms of the scores of the PEASS (Perceptual Evaluation Methods for Audio Source Separation) toolbox, the proposed methods achieve a similar performance as the reference methods. The speech recognition experiments show that the utterances processed by pre-image iterations achieve a consistently better word recognition accuracy than the unprocessed noisy utterances and than the utterances processed by the generalized subspace method.

## Introduction

Speech enhancement is important in the field of speech communications and speech recognition. Many methods have been proposed in the literature (Loizou 2007). Spectral subtractive algorithms were among the first and are probably the simplest (Berouti et al. 1979; Boll 1979). They are based on the assumption that speech and noise are additive and thus the noisy speech signal can be enhanced by subtracting a noise estimate. Usually this is done in frequency domain using the magnitude of the short-time Fourier transform (STFT). For inverse transformation the phase of the noisy signal is considered. Statistical model-based methods provide a framework to find estimates of, e.g., the spectrum or magnitude spectrum of clean speech given the noisy speech spectrum (Ephraim and Malah 1984, 1985; McAulay and Malpass 1980). Subspace methods are based on the assumption that the clean signal only covers a subspace of the Euclidean space where the noisy speech signal exists (Ephraim and Van Trees 1995; Hu and Loizou 2003). Enhancement is performed by separating the noise subspace and the clean speech plus noise subspace and setting the components in the noise subspace to zero. Most speech enhancement algorithms make use of a noise estimate and their performance therefore heavily depends on the quality of the noise estimate. Poor noise estimates may lead to artifacts such as isolated peaks in the spectrum, which are perceived as tones of varying pitch and are known as *musical noise* (Berouti et al. 1979).

Subspace methods make use of principal component analysis (PCA) (Ephraim and Van Trees 1995; Hu and Loizou 2003), which is a linear technique. We therefore investigate if the quality of speech enhancement can be increased by applying a non-linear technique. This leads to the application of kernel methods, which constitute a simple possibility to make linear methods non-linear. Kernel methods transform data samples by mapping them from the input space to the so-called feature space. The non-linear extension of PCA is kernel PCA, which has already been successfully applied in image de-noising (Mika et al. 1999). In (Leitner et al. 2011), we proposed the use of kernel PCA for speech enhancement. Similar to image processing, we apply kernel PCA on patches extracted from the time-frequency representation of speech utterances.

For subspace methods, the number of principal components used for projection is a key parameter for the degree of de-noising. In our framework based on kernel PCA, we empirically observed (see results in Section 6) that the number of used components has almost no influence. We therefore ignore the projection step and only perform the reconstruction step necessary to determine the sample in input space corresponding to the de-noised sample in feature space. We call this *pre-image iterations* (PI) for speech enhancement, as the reconstructed sample in input space is called *pre-image*.

Besides their relation to subspace methods, PI exhibit a similarity to non-local neighborhood filtering (NF) applied for image de-noising (Buades et al. 2005; Singer et al. 2009). While other de-noising algorithms often compute the value of the de-noised pixel solely based on the value of its surrounding pixels, non-local neighborhood filters average over pixels that are located all over the image but have a similar neighborhood. This approach is favorable if images contain repetitive patterns such as textures. Although quite popular for image de-noising, NF has only recently gained attention in the field of speech enhancement. In (Talmon et al. 2011), NF is applied to suppress transient noise bursts. In contrast to our application of PI, NF is not directly applied for de-noising but to gain a noise estimate of the transients that is subsequently used for noise suppression.

In this paper, we compare the performance of kernel PCA and PI for speech enhancement. The variance of the kernel used for the pre-image computation is a tuning parameter that influences the degree of de-noising. Therefore, it has to be adapted according to the SNR. We develop a heuristic method to derive the kernel variance from a noise estimate. This way, PI adapt to different SNRs. Furthermore, an approach for colored noise is developed where the kernel variance is frequency-dependent. The performance of the proposed methods is evaluated in terms of objective speech quality measures and automatic speech recognition results. As objective measures, we employ the perceptual evaluation of speech quality (PESQ) measure (ITU-T 2001) and the scores of the perceptual evaluation of audio source separation (PEASS) toolbox (Emiya et al. 2011). Furthermore we use an automatic speech recognition (ASR) system to measure the performance of noise contaminated and subsequently enhanced data. Note, that the focus here is on evaluating the effects of the enhancement methods and not on optimizing the recognition results per se. Therefore, the speech recognizer is not adapted to the enhanced data.

Experiments are performed on noise corrupted speech from two databases, the *airbone* database and the *Noizeus* database. The utterances are contaminated by additive white Gaussian noise (AWGN) and car noise, respectively, at 0, 5, 10, and 15 dB SNR. As reference, performance results of the generalized subspace method (Hu and Loizou 2003), of spectral subtraction (Berouti et al. 1979), and of the minimum mean-square error (MMSE) log-spectral amplitude estimator (Ephraim and Malah 1985) are provided. In terms of PEASS scores, the proposed methods achieve a similar performance. In terms of word accuracy (WAcc), the utterances enhanced by PI show a significantly higher WAcc than the noisy utterances and the utterances processed by the generalized subspace method.

The paper is organized as follows: In Section 2, we summarize kernel PCA. In Section 3, we describe the application of kernel PCA for speech enhancement. In Section 4, we derive and analyze pre-image iterations and show commonalities to related methods in image and speech processing. In Section 5, we provide implementation details, introduce the used databases, evaluation measures, and the applied speech recognition system. In Section 6, the results are discussed. Section 7 concludes the paper and gives a perspective on future work.

## Kernel PCA

Kernel methods (Bishop 2006) use the map ***Φ*** to transform data samples **x** from the input space  to the feature space

(1) $$\begin{array}{@{}rcl@{}} \boldsymbol{\Phi} : \mathcal{X} \to \mathcal{F} \\ \mathbf{x} \mapsto \boldsymbol{\Phi}(\mathbf{x}), \end{array} $$

where the data is processed. The transformation allows for more flexible algorithms using non-linear mappings. Kernels are defined as inner products between mapped data samples

(2) $$ k(\mathbf{x}_{i},\mathbf{x}_{j}) = \boldsymbol{\Phi}(\mathbf{x}_{i})^{T} \cdot \boldsymbol{\Phi}(\mathbf{x}_{j}).   $$

An important property of kernel methods is that the mapping ***Φ***(**x**) is usually not computed explicitly but only kernels between input samples are evaluated.

Kernel PCA is derived from PCA, which is a widely used technique for dimensionality reduction, lossy data compression, feature extraction, and data visualization. PCA is an orthogonal transformation of the coordinate system of the input data, i.e., the data is projected onto so-called *principal axes*. The new coordinates are called *principal components*. Often the structure in data can be described with sufficient accuracy while using only a small number of principal components. For de-noising, components with low variance are dropped as they are assumed to originate from noise (Mika et al. 1999; Schölkopf and Smola 2002; Schölkopf et al. 1996).

PCA finds the principal axes by diagonalizing the estimated covariance matrix

(3) $$ \mathbf{S} = \frac{1}{M} \sum_{i = 1}^{M} \mathbf{x}_{i} {\mathbf{x}_{i}^{T}}  $$

of a set of *M* data samples $\mathbf {x}_{i} \in \mathbb {R}^{N}$, with *i*=1,…,*M*, assuming zero mean $\sum _{i = 1}^{M} \mathbf {x}_{i} = \mathbf {0}$. This is done by solving the eigenvalue equation

(4) $$ \lambda_{l} \mathbf{u}_{l} = \mathbf{S} \mathbf{u}_{l}  $$

for eigenvalues *λ*_*l*_≥0 and non-zero eigenvectors $\mathbf {u}_{l} \in \mathbb {R}^{N}\setminus \{\mathbf {0}\}$. Substituting (3) into (4) leads to

(5) $$ \lambda_{l} \mathbf{u}_{l} = \frac{1}{M} \sum_{i = 1}^{M} \left({\mathbf{x}_{i}^{T}} \mathbf{u}_{l} \right) \mathbf{x}_{i}.  $$

The product $\left ({\mathbf {x}_{i}^{T}} \mathbf {u}_{l}\right) \mathbf {x}_{i}$ denotes a projection of the eigenvectors **u**_*l*_ with *λ*_*l*_≠0 onto the samples **x**_*i*_. Therefore, following from Equation (5) all eigenvectors lie in the span of **x**_*i*_,…,**x**_*M*_, i.e., all **u**_*l*_ are linear combinations of **x**_*i*_ and can be written as expansions of **x**_*i*_ (Schölkopf and Smola 2002). As PCA is linear, its ability to retrieve the structure within a given data set is limited. If the principal components of variables are non-linearly related to the input variables, a non-linear feature extractor is more suitable. This is realized by kernel PCA (Mika et al. 1999; Schölkopf and Smola 2002).

To derive kernel PCA from standard PCA, let us assume a mapping ***Φ***(**x**) from the input space  to the feature space  as given in (1). As before, we assume that the data is centered in feature space $\sum _{i = 1}^{M} \boldsymbol {\Phi }(\mathbf {x}_{i}) = \mathbf {0}$. In feature space, the estimated covariance matrix is

(6) $$ \mathbf{C} = \frac{1}{M} \sum_{i = 1}^{M} \boldsymbol{\Phi}(\mathbf{x}_{i}) \boldsymbol{\Phi}(\mathbf{x}_{i})^{T}.  $$

To diagonalize the covariance matrix we have to solve the eigenvalue equation

(7) $$ \lambda_{k} \mathbf{v}_{k} = \mathbf{C} \mathbf{v}_{k}  $$

for eigenvalues *λ*_*k*_≥0 and non-zero eigenvectors $\mathbf {v}_{k} \in \mathcal {F}\setminus \{\mathbf {0}\}$, $\mathbf {v}_{k}^{T} \mathbf {v}_{k} = 1$. Equivalently to (5), all eigenvectors **v**_*k*_ that solve this equation lie in the span of ***Φ***(**x**_1_),…,***Φ***(**x**_*M*_). Therefore, each eigenvector **v**_*k*_ can be written as linear combination of the mappings ***Φ***(**x**_*i*_) using the coefficients *α*_*k*1_,…,*α*_*kM*_

(8) $$ \mathbf{v}_{k} = \sum_{i=1}^{M} \alpha_{ki} \boldsymbol{\Phi}(\mathbf{x}_{i}).   $$

Substituting (6) and (8) into (7) leads to

(9) $$ \lambda_{k} \sum_{i = 1}^{M} \alpha_{ki} \boldsymbol{\Phi}(\mathbf{x}_{i}) = \frac{1}{M} \sum_{j = 1}^{M} \boldsymbol{\Phi}(\mathbf{x}_{j})\boldsymbol{\Phi}(\mathbf{x}_{j})^{T} \sum_{i = 1}^{M} \alpha_{ki} \boldsymbol{\Phi}(\mathbf{x}_{i})  $$

for all *k*=1,…,*M*. To enable an expression in terms of kernels we multiply both sides by ***Φ***(**x**_*p*_)^*T*^ such that

(10) $$\begin{array}{@{}rcl@{}}  \lefteqn{\lambda_{k} \sum_{i = 1}^{M} \alpha_{ki} \boldsymbol{\Phi}(\mathbf{x}_{p})^{T} \boldsymbol{\Phi}(\mathbf{x}_{i})}\\ & & =\frac{1}{M} \sum_{j = 1}^{M} \boldsymbol{\Phi}(\mathbf{x}_{k})^{T} \boldsymbol{\Phi}(\mathbf{x}_{j}) \sum_{i = 1}^{M} \alpha_{ki} \boldsymbol{\Phi}(\mathbf{x}_{j})^{T} \boldsymbol{\Phi}(\mathbf{x}_{i}) \end{array} $$

for all *k*=1,…,*M*. Now, let us define an *M*×*M* matrix **K** called *kernel matrix* with the entries

(11) $$ K_{ij} = k(\mathbf{x}_{i}, \mathbf{x}_{j}).   $$

The multiplication of the mappings ***Φ***(**x**_*i*_)^*T*^***Φ***(**x**_*j*_) in (10) can be replaced by a kernel as given in (2) and the equation can be reformulated as

(12) $$ M \lambda_{k} \mathbf{K} \boldsymbol{\alpha}_{k} = \mathbf{K}^{2} \boldsymbol{\alpha}_{k},   $$

where ***α***_*k*_ is the *k*^th^ eigenvector with the entries *α*_*k*1_,…,*α*_*kM*_. The eigenvectors of this system equivalently solve the eigenvalue problem

(13) $$ M \lambda_{k} \boldsymbol{\alpha}_{k} = \mathbf{K} \boldsymbol{\alpha}_{k}.   $$

To find the eigenvectors ***α***_*k*_ the matrix **K** has to be diagonalized. Let us denote the eigenvalues of **K** in the following by *λ*_1_,…,*λ*_*M*_ (which are equivalent to the eigenvalues *Mλ*_*k*_ solving (13)). By requiring a normalization of the eigenvectors in feature space, i.e., $\mathbf {v}_{k}^{T} \mathbf {v}_{k} = 1$, the normalization condition for the eigenvectors ***α***_*k*_ is derived as (Schölkopf and Smola 2002)

(14) $$  1 = \lambda_{k} {\boldsymbol{\alpha}_{k}^{T}} \boldsymbol{\alpha}_{k}.  $$

The projection of a test sample **x** onto the eigenvectors **v**_*k*_ in  can then be determined as

(15) $$ {}\beta_{k} = (\mathbf{v}_{k})^{T} \boldsymbol{\Phi}(\mathbf{x}) = \sum_{i = 1}^{M} \alpha_{ki} \boldsymbol{\Phi}(\mathbf{x}_{i})^{T} \boldsymbol{\Phi}(\mathbf{x}) = \sum_{i = 1}^{M} \alpha_{ki} k(\mathbf{x}_{i},\mathbf{x}).   $$

In summary, to project **x** onto the eigenvectors **v**_*k*_ in  the following steps are required: (i) compute the kernel matrix **K**, (ii) compute its eigenvectors ***α***_*k*_ and normalize them using (13) and (14), (iii) project the data sample **x** using (15).

### 2.1 Centering

Until so far, we have assumed that the data in feature space is centered. This can easily be ensured in input space , but is harder to achieve in feature space , as we usually do not explicitly compute the mapped data and therefore the quantity $\sum _{i=1}^{M} \boldsymbol {\Phi }(\mathbf {x}_{i})$ cannot be assessed. However, as shown in (Schölkopf and Smola 2002; Schölkopf et al. 1996), centering can be done by modifying the kernel matrix **K** such that the *centered kernel matrix*$\tilde {\mathbf {K}}$ is

(16) $$ \tilde{\mathbf{K}} = \mathbf{K} - \mathbf{1}_{M}\mathbf{K} - \mathbf{K} \mathbf{1}_{M} + \mathbf{1}_{M} \mathbf{K} \mathbf{1}_{M},   $$

where **1**_*M*_ is an *M*×*M* matrix with all entries equal to 1/*M*. The eigenvectors ***α***_*k*_ can then be computed by diagonalizing $\tilde {\mathbf {K}}$ instead of **K**.

### 2.2 Kernel PCA for de-noising

To de-noise data, we assume that the directions of eigenvectors corresponding to small eigenvalues only contain information about noise. In contrast, eigenvectors corresponding to large eigenvalues are assumed to contain relevant information, e.g., speech. Therefore, the data sample ***Φ***(**x**) is projected onto the eigenvectors **v**_*k*_ corresponding to the *n* largest eigenvalues while the directions of small eigenvalues are dropped to remove the noise (Mika et al. 1999). To reconstruct the mapping ***Φ***(**x**) after projection we define a projection operator *P*_*n*_ that is given as

(17) $$ P_{n} \boldsymbol{\Phi}(\mathbf{x}) = \sum_{k = 1}^{n} \beta_{k} \mathbf{v}_{k},   $$

where the eigenvectors are assumed to be ordered by decreasing eigenvalue size. Consequently, *P*_*n*_***Φ***(**x**) is a linear combination of the first *n* eigenvectors **v**_*k*_ using the projections *β*_*k*_ of (15) as weights. In case of using all **v**_*k*_, the data sample after projection equals the original data sample *P*_*n*_***Φ***(**x**)=***Φ***(**x**).

The drawback of de-noising in feature space is that in common applications the de-noised data is required in input space. The samples in input space that map to the projected samples in feature space, i.e., the pre-images, are determined by solving the *pre-image problem*.

In the case of applying kernel PCA with a Gaussian kernel

(18) $$ k(\mathbf{x}_{i}, \mathbf{x}_{j}) = \exp{\left(- \frac{\| \mathbf{x}_{i} - \mathbf{x}_{j} \|^{2}}{c} \right)},  $$

where *c* is the kernel variance, one solution for the *pre-image problem* is to approximate the pre-image **z** by minimizing the Euclidean distance *ρ*(**z**) between ***Φ***(**z**) and the projection in feature space *P*_*n*_***Φ***(**x**)

(19) $$ \rho(\mathbf{z}) = \| \boldsymbol{\Phi}(\mathbf{z}) - P_{n} \boldsymbol{\Phi}(\mathbf{x}) \|^{2}.   $$

Mika et al. 1999 showed that for kernels that satisfy *k*(**x**,**x**)=1 for all $\mathbf {x} \in \mathcal {X}$ (such as the Gaussian kernel) the minimization of *ρ*(**z**) can be performed by fixed point iterations. For the Gaussian kernel this results in

(20) $$ \mathbf{z}^{t+1} = \frac{\sum_{i = 1}^{M} \gamma_{i} k(\mathbf{z}^{t}, \mathbf{x}_{i}) \mathbf{x}_{i}}{\sum_{i = 1}^{M} \gamma_{i} k(\mathbf{z}^{t}, \mathbf{x}_{i})},   $$

where **z** is the pre-image, **x**_*i*_ are the original (noisy) samples in input space, *k*(·,·) is the kernel, *t* denotes the iteration index, *M* is the number of samples, and *γ*_*i*_ is given by

(21) $$ \gamma_{i}= \sum_{k = 1}^{n} \beta_{k} \alpha_{ki}  $$

with *β*_*k*_ from (15) and *α*_*ki*_∈***α***_*k*_ in (13). Note that the resulting pre-image **z** is always a linear combination of the input data **x**_*i*_ weighted by the similarity between the pre-image **z** and the data samples **x**_*i*_ and the coefficients *γ*_*i*_. This algorithm is sensitive to initialization which, however, can be tackled by reinitializing with different values.

Several variations of this iterative pre-image solution were proposed. A good overview is provided in (Honeine and Richard 2011). Kwok and Tsang 2004 suggested to use normalized weighting coefficients in (20) to account for centering when using the centered kernel matrix $\tilde {\mathbf {K}}$, i.e.,

(22) $$ \tilde{\gamma}_{i} = \gamma_{i} + 1/M \left(1 - \sum_{m = 1}^{M} \gamma_{m}\right).   $$

Abrahamsen and Hansen 2009 further extended the method by a regularization term

(23) $$ \mathbf{z}_{j}^{t+1} = \frac{\frac{2}{c}\sum_{i = 1}^{M} \tilde{\gamma}_{i} k\left({\mathbf{z}_{j}^{t}}, \mathbf{x}_{i}\right) \mathbf{x}_{i} + \eta \mathbf{x}_{j}}{\frac{2}{c}\sum_{i = 1}^{M} \tilde{\gamma}_{i} k\left({\mathbf{z}_{j}^{t}}, \mathbf{x}_{i}\right) + \eta},   $$

where *η* is a non-negative regularization parameter and **x**_*j*_ is the noisy sample corresponding to the de-noised sample **z**_*j*_. They show that the method is more stable than the method in (Mika et al. 1999).

## Kernel PCA for speech enhancement

**Figure 1:**
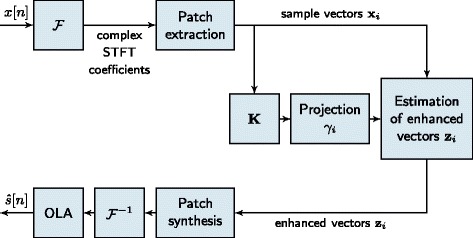
Kernel PCA for speech enhancement.

The application of kernel PCA for speech enhancement is illustrated in the block diagram in Figure 1. To extract feature vectors, i.e., the data samples **x**_*i*_ for kernel PCA, the sequence of STFTs of an utterance is split into so-called frequency bands (see Section 5.1 for details). The frequency bands are decomposed into overlapping patches and the elements in each patch are stacked into **x**_*i*_. One kernel matrix is built from the feature vectors of each frequency band. Each kernel matrix is centered according to (16), then the eigenvalue decomposition (13), normalization of the eigenvectors ***α***_*k*_ (14) and the projection of the data onto the eigenvectors **v**_*k*_ (15) are performed. A Gaussian kernel is used. The pre-images, i.e., the enhanced feature vectors are computed iteratively using normalized iterative pre-imaging (cf. (20) and (22)),

(24) $$ \mathbf{z}_{j}^{t+1} = \frac{\sum_{i = 1}^{M} \tilde{\gamma}_{i} k\left({\mathbf{z}_{j}^{t}}, \mathbf{x}_{i}\right) \mathbf{x}_{i} }{\sum_{i = 1}^{M} \tilde{\gamma}_{i} k\left({\mathbf{z}_{j}^{t}}, \mathbf{x}_{i}\right)},  $$

where $\mathbf {z}_{j}^{t+1}$ is the *j*^t*h*^ enhanced sample within a frequency band at iteration *t*+1, **x**_*i*_ are the noisy samples with *i*=1,⋯,*M*, $\tilde {\gamma }_{i}$ is given by (22) and *M* is the number of samples in the frequency band. We initialize ${\mathbf {z}_{j}^{0}}$ with the noisy sample **x**_*j*_ and iterate (24) until convergence. Finally, the sample vectors are rearranged to patches and the audio signal is synthesized as described in Section 5.1.

## Pre-image iterations for speech enhancement

When subspace methods are applied for speech enhancement, the number of components used for the projection step of PCA is a key parameter. In our framework, we empirically observed that the number of components used for projection has only a minor effect on the outcome of the de-noising process. The de-noising quality is rather the same whether projection is performed on one or more components. De-noising is primarily influenced by the kernel weights and by the value of the kernel variance. Therefore, we completely neglect the projection coefficients $\tilde {\gamma }_{i}$ in (24) by setting them to one.

**Figure 2:**
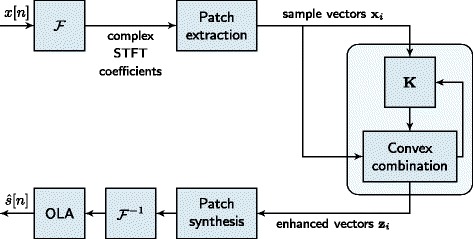
Pre-image iterations for speech enhancement.

The pre-image iteration method is illustrated in the block diagram in Figure 2. The enhanced feature vector **z**_*j*_ is determined as linear combination of the noisy input samples, i.e.,

(25) $$ \mathbf{z}_{j}^{t+1} = \frac{\sum_{i = 1}^{M} k\left({\mathbf{z}_{j}^{t}}, \mathbf{x}_{i}\right) \mathbf{x}_{i}}{\sum_{i = 1}^{M} k\left({\mathbf{z}_{j}^{t}}, \mathbf{x}_{i}\right)}.   $$

The weights of the linear combination are determined by the kernel *k*(·,·), which serves as similarity measure between two samples. The kernel variance *c* is used as parameter to scale the degree to which samples are treated as similar.

We further extended (25) with additional regularization similar as in (Abrahamsen and Hansen 2009) (cf. (23)), such that

(26) $$ \mathbf{z}_{j}^{t+1} = \frac{\frac{2}{c}\sum_{i = 1}^{M} k\left({\mathbf{z}_{j}^{t}}, \mathbf{x}_{i}\right) \mathbf{x}_{i} + \eta \mathbf{x}_{j}}{\frac{2}{c}\sum_{i = 1}^{M} k\left({\mathbf{z}_{j}^{t}}, \mathbf{x}_{i}\right) + \eta},   $$

where **x**_*j*_ is the noisy sample, for which the pre-image should be found and *η*≥0 is the regularization parameter that determines the influence of the noisy sample **x**_*j*_ in PI.

### 4.1 Analysis of pre-image iterations

Pre-image iterations effect de-noising by a linear combination – or weighted average – of noisy feature vectors, where the weights are determined by the kernel. To analyze the de-noising, we define the vector of kernel values

(27) $$ \mathbf{k}_{j} = [k(\mathbf{x}_{j},\mathbf{x}_{1}), k(\mathbf{x}_{j},\mathbf{x}_{2}), \ldots, k(\mathbf{x}_{j},\mathbf{x}_{M})]^{T}  $$

computed between a feature vector **x**_*j*_ and all vectors **x**_*i*_ with *i*=1,…,*M* from one frequency band. This kernel vector always contains one large element equal to one because of self-similarity. The values of the other elements depend on the signal content.

**Figure 3:**
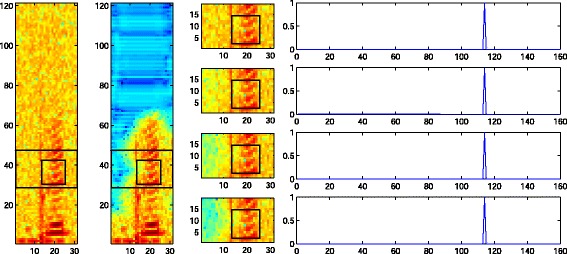
PI in a speech segment shown on the magnitude of the spectral data of the utterance in Figure 8. The columns show from left to right: (i) Noisy segment with one frequency band and a patch marked. The noise level is 10 dB SNR. (ii) Enhanced segment. (iii) The marked frequency band before de-noising and after one to three iterations. (iv) Kernel values between the marked patch and all other noisy patches in the band before de-noising and after one to three iterations. The kernel vector contains only one value significantly larger than zero and no averaging is performed for this patch. Note that the patches are extracted row-wise from left to right and from top to bottom.

If feature vectors with in-phase speech components are compared then the kernel vector contains other elements with larger magnitude. Therefore these feature vectors are combined during PI and noise within these feature vectors is averaged out because it is randomly distributed. In practice and with the described configuration of the feature extraction, there are usually no in-phase feature vectors within a frequency band. Therefore, a feature vector containing speech components is only similar to itself and the noise reduction for this feature vector is limited. This is illustrated in Figure 3. The first and second column represent the noisy magnitude and the enhanced magnitude in a segment where speech is present. The third column shows a frequency band with speech components over several iterations. The marked patch (equivalent to a feature vector) and the corresponding kernel vector in the fourth column do not change during the iterations and no noise reduction is achieved for this patch. This also explains why there is often noise left around speech components and in short speech pauses. To achieve de-noising, smaller patch sizes are necessary. Empirically, we observed, however, that too small patches cause musical noise-like artifacts.

**Figure 4:**
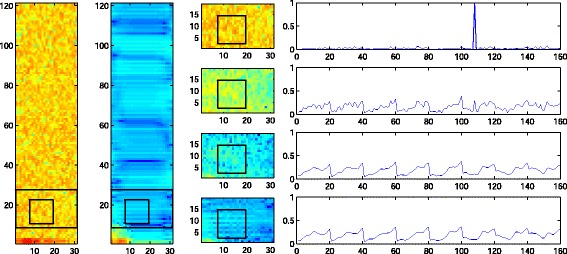
PI in a noisy segment. The columns show from left to right: (i) Noisy segment with one frequency band and a patch marked. (ii) Enhanced segment. (iii) The marked frequency band before de-noising and after one to three iterations. (iv) Kernel values between the marked patch and all other noisy patches in the band before de-noising and after one to three iterations. Note that in contrast to Figure 3, other kernel vector entries besides the entry equal to one are larger than zero and therefore contribute to the averaging in PI.

Feature vectors containing mostly noise exhibit some similarity between all of them. So, in contrast to feature vectors containing speech as shown in Figure 3, there are other kernel values larger than zero besides the kernel value equal to one, as illustrated in Figure 4 in the top right graph. Consequently, in the first iteration several noisy feature vectors are averaged. In the next iteration, the kernel vector is computed between the resulting averaged – or enhanced – feature vector and the original noisy feature vectors. It turns out that the enhanced feature vector is more similar to the noisy feature vectors in terms of similarty measured by the kernel than the original noisy feature vector. Therefore, the kernel vector of the second iteration contains larger elements than the kernel vector of the fist iteration. This can be seen in the graph in the second row and last column in Figure 4. As the kernel values serve as weights for averaging in Equation (25), stronger averaging of feature vectors is performed in the second iteration and the noise is averaged out. This is repeated until the weights are stable and convergence is reached. Note that the feature vectors are complex-valued and that the phase is randomly distributed. Therefore, the feature vectors add up destructively and the noise is canceled.

### 4.2 Relation to non-local neighborhood filtering and to the non-local means algorithm

Performing de-noising on the time-frequency representation of speech incorporates some similarities to methods popular for image de-noising, namely, non-local neighborhood filtering and related methods. In many approaches for image and signal de-noising the de-noised value of the signal is based on neighboring signal values. Gaussian or Gabor filters and anisotropic diffusion are examples for such de-noising approaches.

Most of these methods, however, do not take into consideration one property of many signals and images, namely their *repetitive behavior*, which means that in most signals, patterns of the original noise-free signal occur at different time instances or spatial locations (Singer et al. 2009). For time-domain signals this is the case for every periodic or nearly periodic signal, for instance neuronal spikes or heart beats. In images, there may as well be patches that occur at different spatial locations, e.g., in textures. For de-noising, it is preferable to exploit the occurrence of similar patterns in distant regions of the signal. Instead of using the values in the neighborhood, de-noising is performed over pixels belonging to similar patterns found anywhere in the image. This is realized by NF and bilateral filtering (Barash 2002; Singer et al. 2009). NF is often executed iteratively, as a simple iteration is not sufficient to achieve de-noising. They have a similar iteration scheme as PI (Singer et al. 2009).

The non-local means (NL) algorithm proposed by Buades 2005 is derived from NF. The NL algorithm formulated in vector notation is equivalent to the first iteration of the pre-image iteration equation (25), if the neighborhoods of one pixel are chosen equivalently to patches. A substantial difference, however, is that in the case of speech enhancement the frequency bins – which correspond to the pixels – are complex-valued.

Besides image de-noising, NF has recently been applied in speech enhancement. In (Talmon 2011; Talmon et al. 2011), NF is employed to suppress transient noise. Transient noise consists of short bursts that most speech enhancement algorithms fail to suppress as they are restricted to stationary noise. The repetitive structure of transient noise that causes other enhancement algorithms to be unsuitable for suppression can be exploited by application of non-local filtering. Talmon et al. 2011 noted that the non-local neighborhood filter is equivalent to non-local diffusion filters (NLDF). Although NLDF and pre-image iterations are related, their purpose is considerably different. NLDF make use of a kernel to get reliable estimates of noise transients by constructive averaging. These noise estimates are subsequently used in a speech enhancement algorithm. PI on the other hand use the kernel directly as weight in a linear combination to attenuate noise by destructive averaging of complex-valued feature vectors.

### 4.3 Determination of the kernel variance in PI

As the performance of PI strongly depends on the kernel variance *c*, we adapt *c* for varying noise conditions and levels. Two heuristic approaches are used for the determination of the kernel variance, one for AWGN and one for colored noise (Leitner and Pernkopf 2013). Both make use of a mapping function to derive a suitable value for *c* from a noise estimate.

To find the mapping function, each utterance of the development set is corrupted by noise at different SNRs and PI are applied with different values of *c*. The enhanced recordings are evaluated using the measures of the PEASS toolbox (details about these measures are in Section 5.3.2). As optimization criterion *S* a linear combination of the four scores is used

(28) $$ S = 0.5 \cdot (\text{OPS} + \frac{1}{3} (\text{TPS} + \text{IPS} + \text{APS})).  $$

Additionally, the IPS score has to be greater than 10 to avoid the situation where *S* is large due to good TPS and APS scores but no de-noising is achieved. The noise power is estimated from the beginning of the recording, assuming stationary noise and no speech within this region. The values for *c* that lead to the highest score *S* for the individual utterances and the corresponding noise estimates are fitted by a polynomial of second order. This function is used to obtain values of *c* from noise estimates in the test signals.

For colored noise, a single value for *c* for all frequency bands is insufficient for substantial de-noising as the noise power is not equally distributed over the frequency range. For this reason we derive the averaged noise power estimate for each frequency band individually. These estimates are used in the mapping function derived for white noise to obtain values of *c* for each frequency band. In addition, we derive another mapping function by employing the measured global SNR after enhancement as optimization criterion instead of the score *S*. A comparison showed that the mapping function based on the global SNR results in better de-noising performance.

## Experimental setup and evaluation

To evaluate the proposed speech enhancement algorithms, we performed four different experiments. For all experiments, the speech data was corrupted by noise at 0, 5, 10, and 15 dB SNR. In the first two experiments, we evaluate the results in terms of objective speech quality measures, namely, the PESQ measure and the scores of the PEASS toolbox. In the other two experiments, we compare the performance of a speech recognition system before and after enhancement by PI.

In experiment 1, we compare kernel PCA with the normalized iterative pre-image method (kPCA) as given in (24) and two variants of PI. For the variant denoted by PI_cSNR_, a suitable value for the kernel variance *c* is derived from the performance on a development set for each SNR. For PI with heuristic determination of the kernel variance (PID) the kernel variance is derived from a mapping function as explained in Section 4.3. Enhancement is performed on data of the *airbone* database corrupted by AWGN.

In experiment 2, we perform enhancement on data of the *Noizeus* database corrupted by car noise. We evaluate two variants of PI with frequency-dependent determination of the kernel variance (PIDF) for colored noise. Both variants, PIDF_SNR_ and PIDF_SNR-Var_, employ the SNR to derive the mapping function. Furthermore, the parameter settings of the feature extraction are varied for PIDF_SNR-Var_.

Experiments 3 and 4 use a speech recognition system. In both experiments, data of the *airbone* database is tested. To train the automatic speech recognizer, we use data of the *BAS PhonDat 1* database (Schiel and Baumann 2006). In experiment 3, the data is corrupted by AWGN and enhanced by PI_cSNR_ and PID. In experiment 4, the speech data is corrupted by car noise and enhanced by the PIDF method based on the PEASS scores (PIDF_PEASS_).

### 5.1 Feature extraction and synthesis

**Figure 5:**
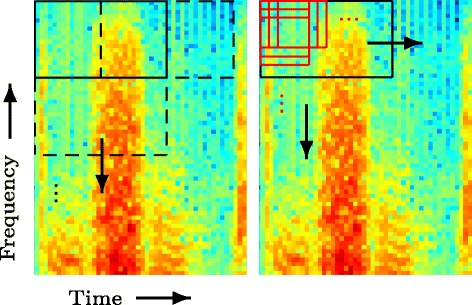
Left hand side: Extraction of frequency bands covering a time range of 10 patches and a frequency range of 8 patches (with 50% overlap along the time axis and no overlap along the frequency axis). Right hand side: Extraction of patches from one frequency band, where the patches cover 12 × 12 bins with an overlap of 10 bins in time and frequency. (Here shown on the clean signal for better visibility).

We use the same feature extraction and synthesis for enhancement by kernel PCA and PI. First the 256-point STFT is computed from frames of 16 ms. The frames have an overlap of 50% and a Hamming window is applied. The resulting time-frequency representation is split into time segments of 0.25 ms. Each segment is split on the frequency axis to reduce computational costs which results in so-called *frequency bands*. Sample vectors are retrieved from these frequency bands by first extracting quadratic patches in an overlapping manner, where the size of each patch is 12×12 with an overlap of 11. This is illustrated in Figure 5. On the left hand side, frequency bands are marked as black rectangles, on the right hand side, quadratic patches within one frequency band are marked as red squares. In previous experiments, windowing of the patches was beneficial, so a 2D Hamming window is applied. Then, the values in the patches are re-ordered in column-major order to form the sample vectors $\mathbf {x}_{i} \in \mathbb {C}^{144}$. The frequency bands cover a frequency range corresponding to 8 patches (i.e., 19 bins) and a time range corresponding to 20 patches (i.e., 31 bins). Along the frequency axis bands have an overlap of 50% or no overlap – depending on the experiment – and along the time axis the overlap is 10 patches. This configuration was chosen due to good empirical results. After processing, the enhanced audio signal is synthesized by reshaping the enhanced sample vectors **z**_*i*_ to patches. The patches of all frequency bands belonging to one time segment are rearranged using the overlap-add method with weighting as described in (Griffin and Lim 1984), generalized for the 2D domain. Then, the STFT bins of overlapping time segments are averaged, the inverse Fourier transform is applied on the bins of each frame and the audio signal is synthesized with the weighted overlap-add method in (Griffin and Lim 1984).

### 5.2 Databases

#### 5.2.1 Noizeus database

The *Noizeus* database was proposed to enable the comparison of speech enhancement methods (Hu and Loizou 2007). The database contains recordings of 30 IEEE sentences (in English) (IEEE Subcommitee 1969), spoken by three female and three male speakers (five sentences each). The sentences were recorded with 25 kHz sampling frequency and downsampled to 8 kHz. Furthermore, the speech signals were filtered by the modified Intermediate Reference System filters used in ITU-T P.862 (ITU-T 2001) to simulate the frequency characteristics of a telephone handset. The recordings are corrupted by eight types of real-world noise. The SNR computation is based on the active speech level (ASL) (ITU-T 2011). We use the data corrupted by car noise and additionally contaminated clean recordings by AWGN for the derivation of the mapping functions. The development set contains one sentence per speaker and SNR condition.

#### 5.2.2 Airbone database

The *airbone* database consists of 120 utterances read by six speakers – three male and three female – of the Austrian variety of German (Domes 2009). The utterances are recorded by the close-talk microphone of a headset with a sampling frequency of 16 kHz. The headset is further supplied with a bone conduction microphone, hence the name *airbone* database. The signal of the bone microphone, however, is not used in this work. The data is corrupted by AWGN and by car noise from the *NOISEX-92* database (Varga and Steeneken 1993) with consideration of the ASL. A subset of two utterances per speaker and SNR condition is used for development, i.e., for setting the kernel variance or for deriving the mapping function for estimating the kernel variance.

#### 5.2.3 BAS PhonDat 1 database

The *BAS PhonDat 1* (BAS PD1) database belongs to the *Bavarian Archive for Speech Signals Corpora* (Schiel and Baumann 2006). The BAS PD1 corpus contains read speech uttered by 201 different speakers of German. In total, 21587 utterances were recorded with a sampling frequency of 48 kHz. The data was downsampled to 16 kHz.

We use 4999 clean utterances of the BAS database to train the speech recognizer. These utterances correspond to 50 different speakers resulting in around 100 utterances per speaker and 1504 different words in total. The main reason to use the data of the BAS database is that the *airbone* database initially used for speech enhancement does not provide a sufficient amount of data for training. However, this way the effect of presenting unseen data to a speech recognizer can optimally be studied.

### 5.3 Objective quality measures

For objective evaluation we use two measures:

#### 5.3.1 PESQ

The PESQ measure is recommended by the ITU-T for quality assessment of narrow-band telephone speech and narrow-band speech codecs (ITU-T 2001; Rix et al. 2001). The PESQ measure returns a mean opinion score (MOS) between 0.5 and 4.5. In (Hu and Loizou 2008), PESQ was reported to show high correlation with the outcome of subjective listening tests on speech enhancement algorithms.

#### 5.3.2 PEASS

The objective measures of the PEASS toolbox are developed for audio source separation (Emiya et al. 2011). The design of these measures is based on the outcome of subjective listening tests and the measures strongly agree with subjective scores. With the PEASS toolbox four aspects of the signal can be tested: the global quality (OPS - overall perceptual score), the preservation of the target signal (TPS - target perceptual score), the suppression of other signal (IPS - interference perceptual score), and the absence of additional artificial noise (APS - artifact perceptual score). The scores range from 0 to 100, larger values denote better performance.

### 5.4 Automatic speech recognition

The automatic speech recognizer is based on the *Hidden Markov Toolkit* (HTK) (Young et al. 2006). The front-end (FE) and the back-end (BE) are both derived from the standard recognizer of the Aurora-4 database (Hirsch 2002). The FE computes Mel frequency cepstral coefficients (MFCCs) by using a sampling frequency of 16 kHz, a frame shift of 10 ms, a window length of 32 ms, 1024 frequency bins, 26 Mel channels, and 13 cepstral coefficients. Cepstral mean normalization is employed on the MFCCs. Furthermore, delta and delta-delta features are computed with a window length of 5 (half length 2). This finally leads to a feature vector of 39 components.

For training, the BE uses a dictionary based on 34 SAMPA-monophones. For each triphone, a hidden Markov model (HMM) is trained, which consists of 6 states and Gaussian mixture models of 8 components per state. To reduce the complexity and to overcome the lack of training data for some triphones, a tree-based clustering based on monophone-classification is applied. The grammar used for training is probabilistically modeled. In contrast to that, a rule-based grammar is applied for testing as the utterances of the *airbone* database obey very strict grammar rules.

The ASR experiments are evaluated in terms of word accuracy, which is defined as

(29) $$ \text{WAcc} = \frac{N - S - D - I}{N} \times 100 \%,  $$

where *N* is the number of words, *S* is the number of substitutions, *D* is the number of deletions and *I* is the number of insertions.

In addition to the WAcc, we evaluated if the performance difference between the pre-image iteration methods and the reference methods is statistically significant. We use a *matched pairs test* as recommended in (Gillick and Cox 1989). The matched pairs test is based on the pair-wise comparison of the recognition rates on the same utterance processed by two different algorithms. This test is suitable to test the significance of ASR results on speech segments that are statistically independent, i.e., an error in one segment is not influenced by an error in a preceding segment. This is the case for the experiments on the *airbone* database, as we test utterances independent from each other. For all evaluations, we employ a significance level of 0.01.

## Results and discussion

In this section, we present the evaluation results of the experiments described in Section 5. As a benchmark, results of the generalized subspace method (Hu and Loizou 2003), spectral subtraction (Berouti et al. 1979), the MMSE log-spectral amplitude estimator (Ephraim and Malah 1985), and of the noisy baseline are given.

### 6.1 Experiment 1: Kernel PCA, PI with SNR-dependent kernel variance, and PI with heuristic determination of the kernel variance

**Figure 6:**
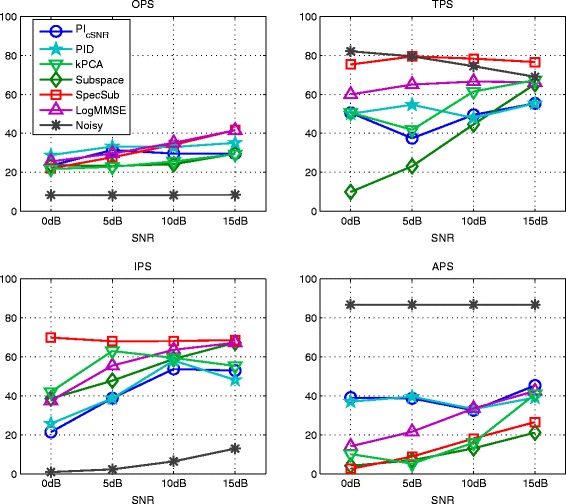
Results of kernel PCA with normalized pre-imaging (kPCA), PI with SNR-dependent setting of the kernel variance (PI_cSNR_), the generalized subspace method (Subspace), spectral subtraction (SpecSub), and the MMSE log-spectral amplitude estimator (LogMMSE) in terms of overall perceptual score (OPS), target perceptual score (TPS), interference perceptual score (IPS), and artifact perceptual score (APS) on the test set of the *airbone* database corrupted by AWGN.

**Figure 7:**
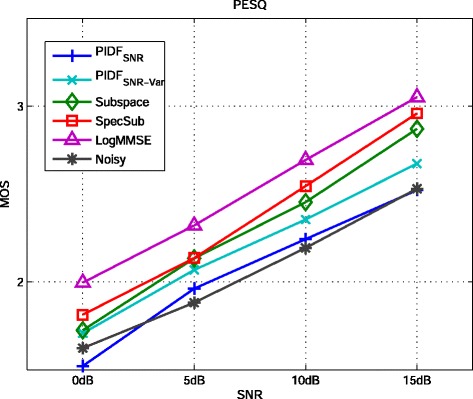
Results of kernel PCA with normalized pre-imaging (kPCA), PI with SNR-dependent setting of the kernel variance (PI_cSNR_), the generalized subspace method (Subspace), spectral subtraction (SpecSub), and the MMSE log-spectral amplitude estimator (LogMMSE) in terms of the PESQ measure on the test set of the *airbone* database corrupted by AWGN.

Figure 6 and Figure 7 show the results of kernel PCA with normalized iterative pre-image computation (kPCA) as given in Equation (24), of PI with SNR-dependent setting of the kernel variance (PI_cSNR_), and of PI with heuristic determination of the kernel variance (PID). For kPCA and PI_cSNR_, the choice of a suitable value for the kernel variance and the regularization parameter *η* is based on the performance in terms of the PEASS scores on the development set. For both methods, the values for *c* are 6, 3.5, 0.75, and 0.2 for 0, 5, 10, and 15 dB, respectively. For kPCA, no regularization is applied. For PI_cSNR_, the regularization parameter *η* is set to 0.5 for all SNR conditions and for PID to 0.25 for 0 dB SNR and 0.75 for the other SNRs.

All methods gain an improvement of overall quality (OPS) in comparison to the noisy speech data. The performance of PI_cSNR_ and PID is superior to the performance of kPCA and the generalized subspace method. For low SNRs, the OPS of PI_cSNR_ is similar to spectral subtraction and the MMSE log-spectral amplitude estimator, while for high SNRs the other methods are superior. The performance of PID is better than the reference methods in low SNRs. It is worth noting that the APS for the PI_cSNR_ and PID is better than for the other methods in most SNR conditions, indicating that there are few artifacts such as, for instance, musical noise in the case of the generalized subspace method and spectral subtraction.

Figure 7 shows the PESQ. All methods improve the score in comparison to the noisy speech data, except of PID at 15 dB SNR. This indicates that the used mapping function is not optimally chosen at high SNRs. Similar as for the OPS, the performance of PID is better than the performance of kPCA and PI_cSNR_. In low SNRs, the score of PID is similar to the reference methods, while it is lower in high SNRs. This also suggests that the mapping function for high SNRs is not optimal. The presence of musical noise in the recordings enhanced by spectral subtraction and the generalized subspace method is not reflected by the PESQ measure.

Listening to the signals enhanced by the proposed methods reveals that noise is removed and no musical noise occurs^a^. However, there is some background noise left around speech components, which is also reflected by the rather low IPS of the pre-image iteration methods. In the case of kPCA, a buzz-like artifact can be perceived. Note that this is well reflected by the low APS.

**Figure 8:**
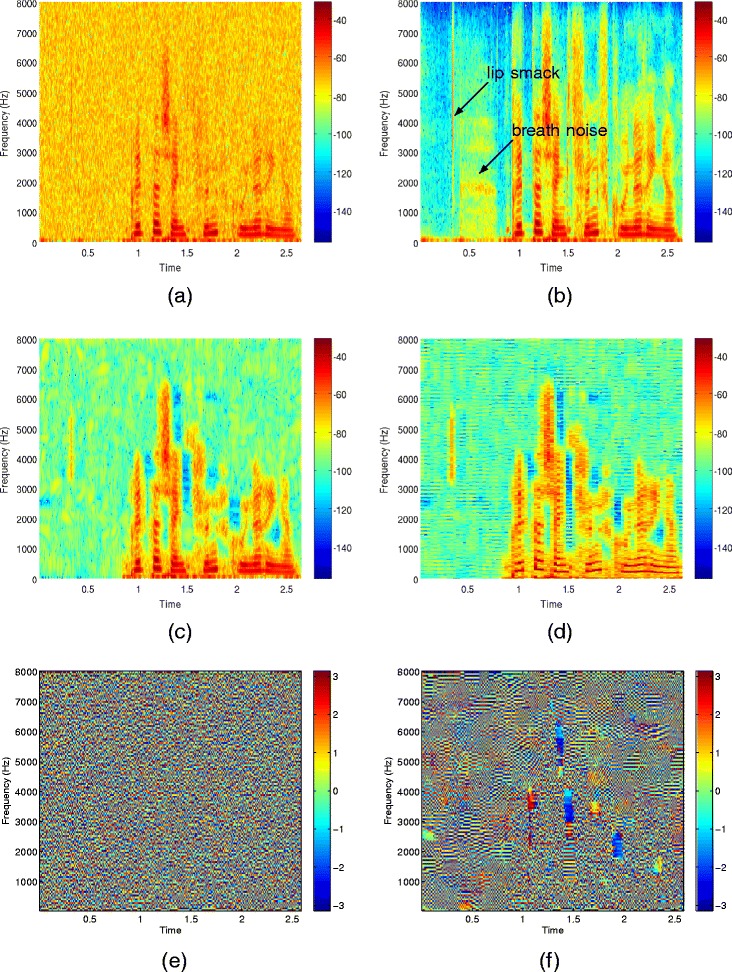
The utterance “Britta schenkt fünf grüne Ringe.” produced by a female speaker of the *airbone* database. Note that the beginning is free of speech but contains a lip smack and breath noise. Spectrogram of the **(a)** signal corrupted by additive white Gaussian noise at 10 dB SNR, **(b)** clean signal, **(c)** signal enhanced by the kernel PCA method, and **(d)** enhanced by kernel PCA and plotted with higher frequency resolution. **(e)** phase of the noisy signal, **(f)** phase after kernel PCA. The pattern visible in the phase plot **(f)** causes the harmonic artifacts in **(d)**.

Figure 8 shows the spectrograms of an utterance of the *airbone* database. The utterance is spoken by a female speaker and has been corrupted by AWGN at 10 dB SNR. Figure 8(a) and (b) show the spectrograms of the corresponding noisy and clean signal, respectively. Figure 8(c) shows the spectrogram after enhancement by kernel PCA. Looking at the spectrogram with a higher frequency resolution in Figure 8(d) shows that the artifacts correspond to harmonics that smoothly change over time. The frequency of the artifact is related to the number of Fourier coefficients used for the STFT. Figure 8(e) and (f) show a plot of the phase before and after enhancement. After enhancement, a regular structure structure is visible. This originates from samples that converge to the same solution within one frequency band and causes the buzz-like artifact in Figure 8(d).

**Figure 9:**
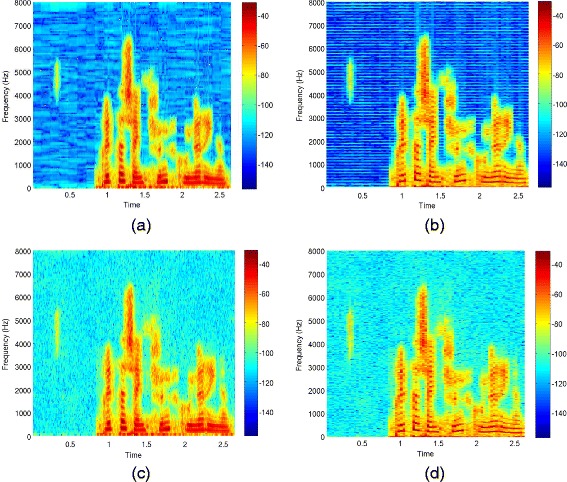
Spectrograms after enhancement by pre-image iterations **(a)** without regularization plotted with low frequency resolution, **(b)** without regularization plotted with high frequency resolution, **(c)** with regularization plotted with low frequency resolution and **(d)** with regularization plotted with high frequency resolution. Note that there is still a harmonic artifact in **(b)**, however, its magnitude is lower than in the case of kernel PCA and hence it cannot be perceived. With regularization there is more remaining noise than without but this can as well not be perceived. Furthermore the harmonic artifact is masked by this residual noise, as can be seen in **(d)**.

The spectrogram of PI with regularization in Figure 9 (a) shows that there are fewer artifacts in comparison to kernel PCA in Figure 8 (c). Figure 9 (b) shows the spectrogram of PI without regularization at a higher frequency resolution. It can be seen that there is still a harmonic artifact, however, its magnitude is considerably lower than in the case of kernel PCA. With regularization this artifact is additionally masked. Listening to the utterance confirms that the artifact cannot be perceived. With regularization in (26), the audio signal sounds similar as without regularization but with slightly more background noise that changes with the value of *η*. The different levels of background noise are caused by the weighting of the noisy samples by *η* in the regularization term.

### 6.2 Experiment 2: PI with frequency-dependent determination of the kernel variance for colored noise

**Figure 10:**
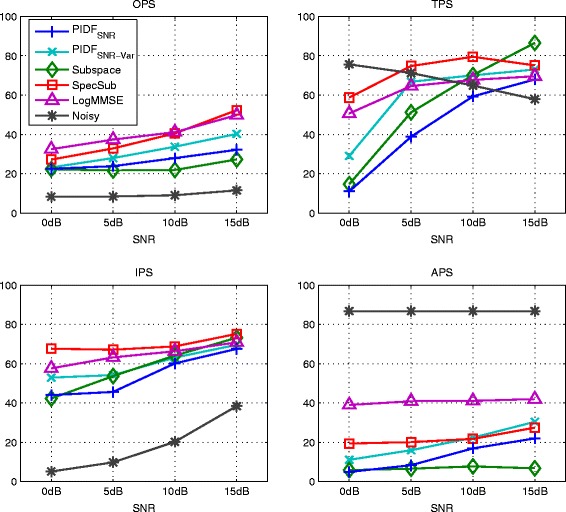
Results of PI with frequency-dependent determination of the kernel variance based on the SNR (PIDF_SNR_), with additional variation of the feature extraction parameters (PIDF_SNR-Var_), of the generalized subspace method (Subspace), spectral subtraction (SpecSub), and the MMSE log-spectral amplitude estimator (logMMSE) in terms of PEASS scores on the test set of the *Noizeus* database corrupted by car noise.

**Figure 11:**
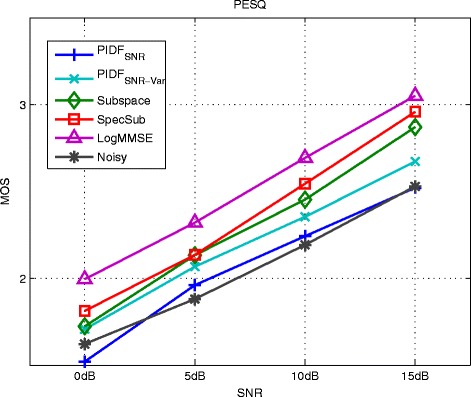
Results of PI with frequency-dependent determination of the kernel variance based on the SNR (PIDF_SNR_), with additional variation of the feature extraction parameters (PIDF_SNR-Var_), of the generalized subspace method (Subspace), spectral subtraction (SpecSub), and the MMSE log-spectral amplitude estimator (logMMSE) in terms of the PESQ measure on the test set of the *Noizeus* database corrupted by car noise.

Figure 10 and 11 show the results of the PIDF methods based on the global SNR as optimization criterion (PIDF_SNR_ and PIDF_SNR-Var_). For the PIDF_SNR-Var_ method, the size of frequency bands in the feature extraction step was modified to a length of 0.4 seconds and a height of 3 patches as this improved the results in comparison to the standard parametrization of 0.25 seconds length and 8 patches height.

The overall quality of PIDF_SNR_ and PIDF_SNR-Var_ is better than the overall quality of the noisy signal and the generalized subspace method, however, lower than the overall quality of the other reference methods. PIDF_SNR-Var_ achieve consistently higher scores than PIDF_SNR_. In terms of PESQ, the reference methods show superior performance, but the difference is rather small.

Listening to the signals enhanced by the PIDF methods reveals that there is noise left around speech components. For PIDF_SNR-Var_ the noise components are smoother than for PIDF_SNR_, however, a hum can be perceived in the background. This is similar to the buzz-like artifact and caused by the smaller number of feature vectors in one frequency band due to the changed configuration. In the signals processed by the MMSE log-spectral amplitude estimator there is some background noise left and minor musical noise-like artifacts can be perceived, while the signals enhanced by spectral subtraction and the generalized subspace method are strongly affected by musical noise.

### 6.3 Experiment 3: ASR of data corrupted by white noise and enhanced by PID

Table 1 shows the WAcc for PI_cSNR_ and for PID tested on the *airbone* database. Table 2 shows the results of the statistical significance test between PID and the reference methods. We used the matched pairs test which is based on the pair-wise comparison of the recognition rates on the same utterance processed by two algorithms. The difference of errors is computed for each pair and the mean of differences is tested with respect to equality to zero. A mean different from zero indicates a statistical difference of the WAcc of two algorithms. For all evaluations, we employ a significance level of 0.01.

**Table 1:** **WAcc on data corrupted by AWGN before and after enhancement**

**Condition**	**0 dB**	**5 dB**	**10 dB**	**15 dB**	**Average**
Noisy	0.00	15.56	38.89	65.56	30.00
PI_cSNR_	27.22	53.89	68.33	72.59	57.15
PID	35.93	58.70	72.22	77.59	61.11
Subspace	2.59	4.63	16.30	42.96	16.62
Subspace_MNS_	22.96	36.48	46.85	68.89	43.80
SpecSub	25.74	53.15	73.89	85.56	59.59
LogMMSE	37.78	58.15	74.63	89.07	64.91
Clean	97.78

**Table 2:** **Results of the statistical significance test between PID and the reference methods for the WAcc in Table** 1

**PID**	**0 dB**	**5 dB**	**10 dB**	**15 dB**
Noisy	*	*	*	*
Subspace	*	*	*	*
SpecSub	*			-
LogMMSE	-		-	-

The asterisk indicates a significantly better performance of PID with a significance level of 0.01, while the minus sign indicates a lower performance.

The WAcc for the noisy data clearly states that the recognizer performance suffers from the noise contamination. The enhancement based on PI successfully increases the WAcc in comparison to the noisy data. The WAcc of the PID is always superior to the WAcc of the generalized subspace method, similar to the WAcc of spectral subtraction and lower than the WAcc of the MMSE log-spectral amplitude estimator. The superior performance of PID is significant for the generalized subspace method, for spectral subtraction at 0 dB SNR and the noisy data. The relatively high WAcc of the pre-image iteration methods shows a different trend compared to the PESQ results, where the scores of the reference methods are better than for the pre-image iteration methods. The comparison of PI_cSNR_ to PID reveals that PID always achieve higher word accuracies. This confirms that the heuristic determination of the kernel variance is preferable over using a fixed value for one noise condition.

**Figure 12:**
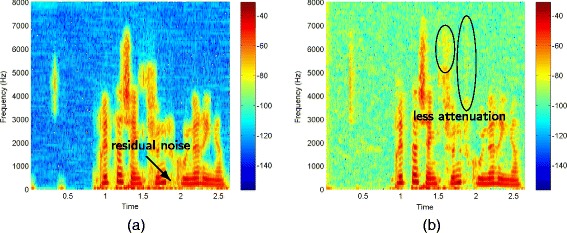
Comparison of the spectrograms after application of **(a)** PID and **(b)** the MMSE log-spectral amplitude estimator on the example utterance corrupted by AWGN at 15 dB SNR. The MMSE log-spectral amplitude estimator removes noise near speech components more efficiently and performs less attenuation on low energy speech components.

Listening to the utterances processed by the generalized subspace method and by spectral subtraction reveals that musical noise is very prominent. The utterances enhanced by the pre-image iteration methods and the MMSE log-spectral amplitude estimator are less affected by such artifacts. This explains the better performance of pre-image iteration methods and the MMSE log-spectral amplitude estimator, especially in low SNR conditions. The MMSE log-spectral amplitude estimator is outperforming the pre-image iteration methods. One reason is that the PI methods attenuate speech components in low energy speech regions. Another reason is that PID leave more residual noise near speech components than the MMSE log-spectral amplitude estimator. Figure 12 illustrates both effects for the example speech utterance corrupted by AWGN at 15 dB SNR, for which the difference in WAcc is the largest. The performance of PID could be improved by tuning the kernel variance of different frequency bands such that high frequency bands are less attenuated than low frequency bands. This would be more natural as the energy of speech decreases with increasing frequency.

To test the hypothesis that musical noise is problematic for the speech recognizer we further evaluated the WAcc on data corrupted by AWGN, enhanced by the generalized subspace method and subsequently post-processed by the musical noise suppression (MNS) method proposed in (Leitner and Pernkopf 2012). The results are included in Table 1 and denoted as Subspace_MNS_. The WAcc is better after the MNS and the performance difference is significant. Hence, the musical noise is indeed a problem for the recognizer and speech enhancement methods introducing too many artifacts may be counterproductive, as shown for the generalized subspace method, where the WAcc is even lower than the WAcc for the noisy data.

### 6.4 Experiment 4: ASR of data corrupted by colored noise and enhanced by PIDF

Table 3 shows the WAcc after enhancement by the PIDF method for colored noise. In the presented experiments car noise was used. The mapping function is based on the PEASS scores, hence the results are denoted by PIDF_PEASS_. Table 4 shows the results of the statistical significance test between PIDF_PEASS_ and the reference methods.

**Table 3:** **Wacc on data corrupted by car noise before and after enhancement**

**Condition**	**0 dB**	**5 dB**	**10 dB**	**15 dB**	**Average**
Noisy	1.30	25.93	62.78	85.19	43.80
PIDF_PEASS_	34.95	62.04	81.48	89.26	66.93
Subspace	8.52	27.04	66.85	81.48	45.97
SpecSub	29.26	61.11	79.26	90.74	65.23
LogMMSE	52.78	75.74	86.11	94.07	77.17
Clean	97.78

**Table 4:** **Results of the statistical significance test between PIDF and the reference methods for the WAcc in Table** 3

**PIDF**_**PEASS**_	**0 dB**	**5 dB**	**10 dB**	**15 dB**
Noisy	*	*	*	
Subspace	*	*	*	*
SpecSub	*			
LogMMSE	-	-	-	-

The asterisk indicates a significantly better performance of PIDF with a significance level of 0.01, while the minus sign indicates a lower performance.

The results for the experiments with car noise show that this type of noise is less harmful to the performance of the recognizer than white noise. This can be explained by the fact that the noise energy is concentrated below 1kHz, where the speech components are relatively strong and the distortion by the noise therefore is limited. Similar to the experiments with white noise, the WAcc of PIDF_PEASS_ is higher than the WAcc of the generalized subspace method, similar to the WAcc of spectral subtraction and lower than the performance of the MMSE log-spectral amplitude estimator. The performance is significantly better in comparison to the noisy data except for 15 dB, better than the generalized subspace method and than spectral subtraction for 0 dB.

**Figure 13:**
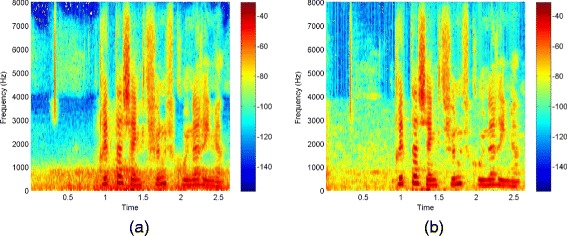
Comparison of the spectrograms after application of **(a)** PIDF and **(b)** the MMSE log-spectral amplitude estimator on the example utterance corrupted by car noise at 5 dB SNR. The MMSE log-spectral amplitude estimator removes noise more efficiently in low frequency regions.

The difference between PIDF and the MMSE log-spectral amplitude estimator is illustrated in Figure 13 for 5 dB SNR. The superior performance of the MMSE log-spectral amplitude estimator can be explained by the better de-noising in low frequency regions. For PIDF there is more residual noise left. To overcome this, the derivation of the kernel variance should be refined: either by applying a finer resolution of the frequency bands (as explained in Section 5.1) or by a tuning factor that adapts the frequency bins within one band. This enables to apply higher attenuations on bins in low frequency regions, where speech components have more energy, and lower attenuation in high frequency regions, where speech components are weaker. This is investigated in future work.

## Conclusion

In this paper, we used kernel PCA for speech enhancement. We apply kernel PCA on complex-valued feature vectors extracted from the time-frequency representation of noisy utterances and make use of an iterative pre-image method to synthesize the de-noised audio signal.

Experimental results show that for the iterative pre-image methods the weighting factor derived from the projection of kernel PCA only contributes little to de-noising. The de-noising mainly results from the linear combination of complex-valued feature vectors, which leads to cancellation of random-phase noise components. We therefore simplify the pre-image computation by setting the weighting coefficients to one and call this *pre-image iterations* for speech enhancement. Both kernel PCA and PI depend on the kernel variance as tuning parameter, which influences the degree of de-noising. We therefore extended PI by heuristic determination of the kernel variance for white noise and by frequency-dependent determination of the kernel variance for colored noise. This way, PI adapt to arbitrary noise conditions.

The evaluation in terms of PESQ and PEASS shows that the performance of kernel PCA and PI for speech enhancement is comparable to the performance of the reference methods in low SNRs, while in high SNRs spectral subtraction and the MMSE log-spectral amplitude estimator achieve better scores. We further evaluated the effect of speech enhancement on automatic speech recognition. The word accuracies on speech enhanced by PI are superior to the word accuracies achieved on noisy speech and by the generalized subspace method. In contrast to PI, the generalized subspace method is prone to musical noise, which deteriorates the recognition performance. The recognition performance for the MMSE log-spectral amplitude estimator is better than the performance of PI, while the performance for spectral subtraction is similar.

In future, we would like to extend the pre-image iteration method by a noise tracker to generalize the method from stationary noise to other noise types such as babble noise. Furthermore, we plan to build a recognizer for data of the Noizeus database for speech enhancement.

## Endnote

^a^Audio samples are provided on http://www2.spsc.tugraz.at/people/chrisl/audio/springer2015.
